# Supplementary material for: Systematic Analysis of Clemastine, a Candidate Apicomplexan Parasite-Selective Tubulin-Targeting Agent
Source: Int J Mol Sci. 2021 Dec 22;23(1):68. doi: 10.3390/ijms23010068 (PMC8744746; doi:10.3390/ijms23010068)
Supplement: Supplementary file 1 [file ijms-23-00068-s001.zip › ijms-1500520-supplementary.pdf]

International Journal of *Molecular Sciences*

Supplementary Materials

Systematic Analysis of Clemastine, A Candidate Apicomplexan 2 Parasite-Selective Tubulin-Targeting Agent

Izra Abbaali [1], Danny Truong [1], Shania Day [1], Nancy Haro-Ramirez [1], and Naomi Morrissette [1,*]

1. Supplementary Figures

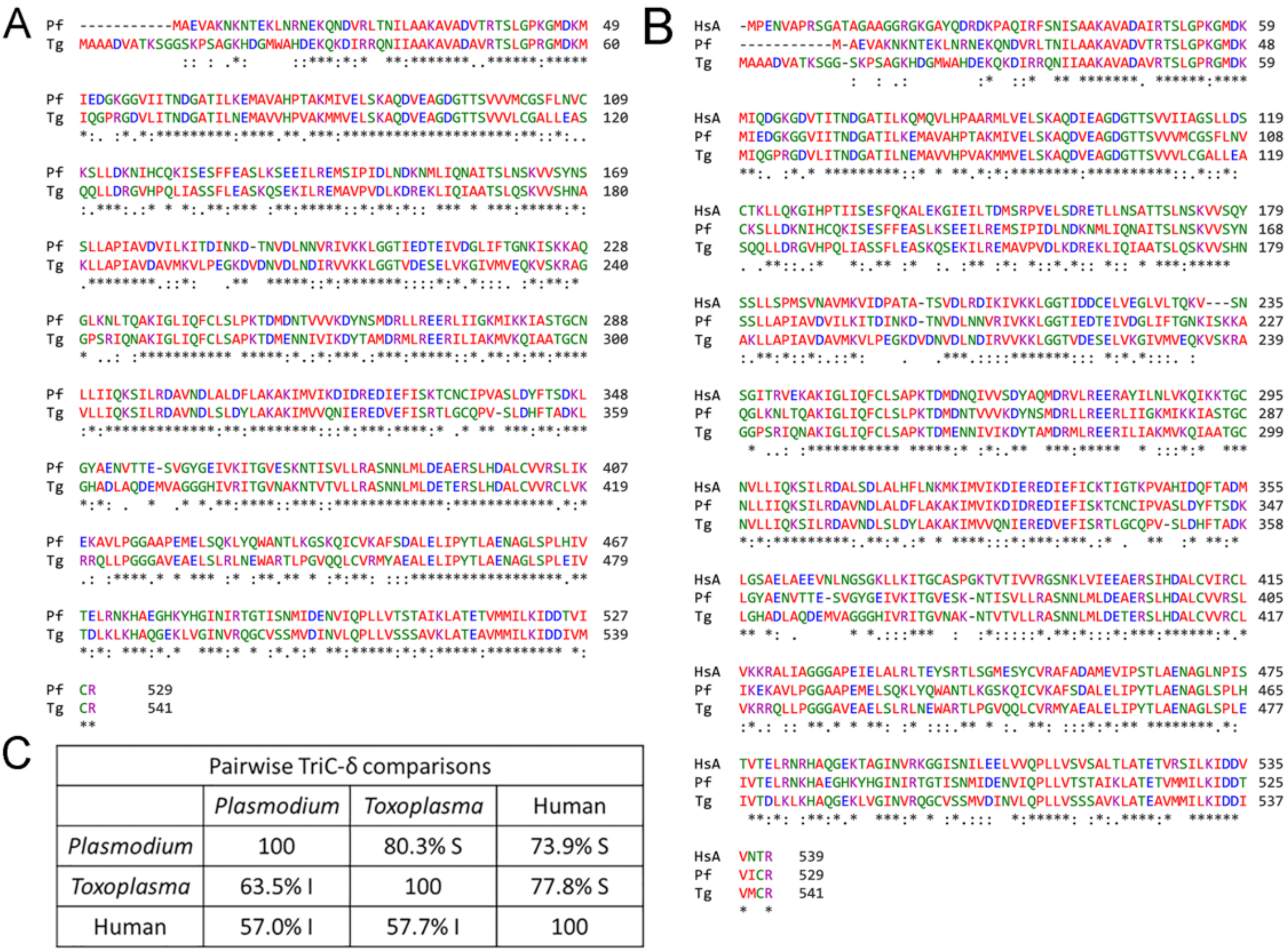

Figure S1. Clustal omega alignments of the tubulin chaperone TRiC subunit from *Toxoplasma* (Tg) and *Plasmodium falciparum* (Pf) (**A**) or *Toxoplasma*, *Plasmodium* and human (Hs) proteins (**B**). Amino acids are colored according to their physicochemical properties: small and hydrophobic residues are red, acidic are blue, basic are magenta, and the remaining amino acids are colored green. (**C**) Pairwise comparisons performed with EMBOSS Needle [47] indicate that *Toxoplasma* and *Plasmodium* TRiC-δ proteins have greater identity (I) and similarity (S) than either parasite protein.

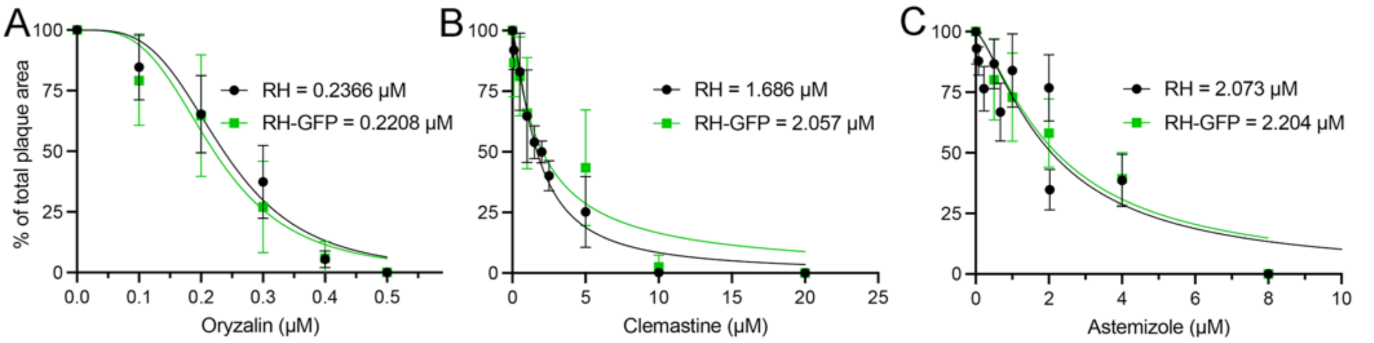

Figure S2. Plaque-based EC_{50} assays measure inhibition of lytic growth. The relationship between plaque area and drug concentration is graphed to determine the 50% inhibitory concentration with values normalized to vehicle control cultures. The values for oryzalin (**A**), clemastine (**B**) and astemizole (**C**) show that all exhibit antiparasitic activity in the micromolar range and are comparably active at inhibiting both RH strain parasites and a GFP-expressing RH-derived line. All results represent the average of at least 9 readings (3 biological replicates, each with 3 technical replicates) ± standard deviation.

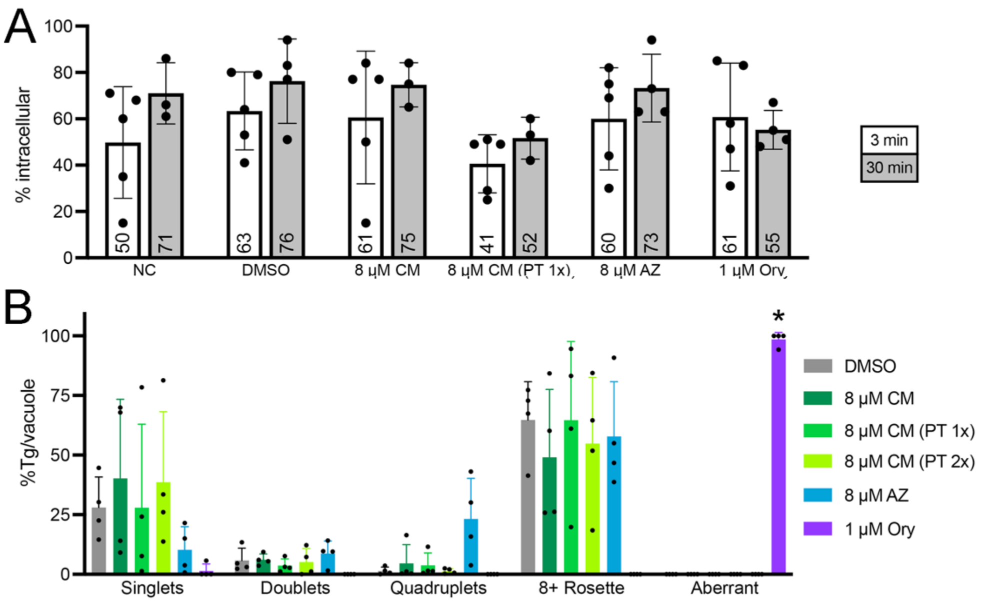

Figure S3. Lytic growth consists of invasion, replication, and egress. (**A**) GFP-expressing RH strain tachyzoites are added to HFF cells in control or drug-containing media for 3-30 minutes. After fixation subsequent antibody staining without permeabilization distinguishes intracellular tachyzoites from extracellular (host cell attached) parasites, which are detected by staining for the externally exposed SAG-1 surface antigen detected with a red-fluorescing secondary antibody. Statistical analyses were run using an ordinary two-way ANOVA. The results represent the average of 30 readings (3 biological replicates with 10 fields of view for each treatment) ± standard deviation. (**B**) Intracellular *Toxoplasma* parasites within membrane-bound vacuoles replicate by endodyogeny. RH strain tachyzoites double every 60 minutes and replication within a vacuole is largely synchronous, leading to exponential expansion of parasites. Live imaging of GFP-expressing tachyzoites permits detection of differences in replication rate and overt changes in parasite morphology or differentiation state. No delays in replication are detected at 36 hours for clemastine and astemizole; oryzalin treated parasites rapidly transform to aberrant forms. Data are displayed as the fraction of total vacuoles/field that contain one, two, four, or eight or more (8+) parasites. Statistical analyses were run using a two-way ANOVA. The results represent the average of 40 readings (4 biological replicates with 10 fields of view for each treatment) ± standard deviation.

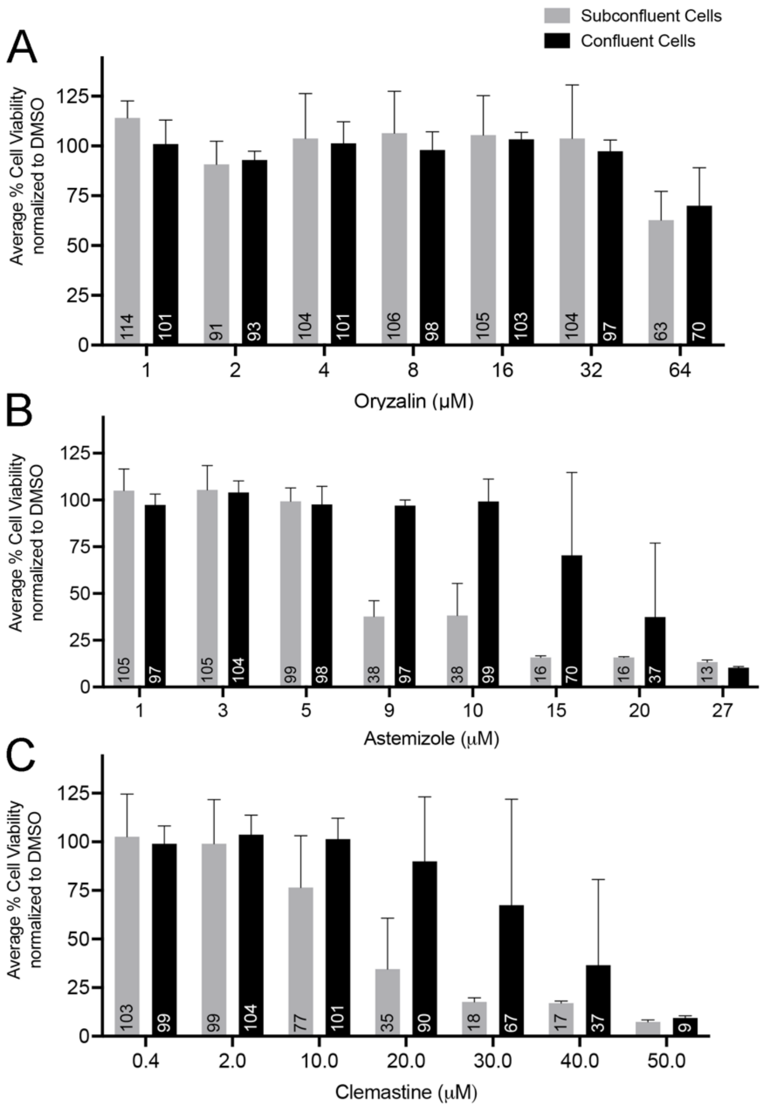

Figure S4. MTT assay-based quantification of compound toxicity to human fibroblasts. Because replicating human fibroblasts are likely to be more sensitive to compounds than contact-inhibited confluent monolayers, we evaluated the effects of oryzalin (**A**), astemizole (**B**), and clemastine (**C**) on both replicating (light gray) and confluent (dark gray) monolayers of HFF cells. In all compounds, subconfluent cultures showed greater compound sensitivity. All results represent the average of at least 9 readings (3 biological replicates, each with 3 technical replicates) ± standard deviation. .
